# Incidence, individual, and macro level risk factors of severe binocular visual impairment and blindness in persons aged 50 and older

**DOI:** 10.1371/journal.pone.0251018

**Published:** 2021-05-03

**Authors:** Daniel Kreft, Gabriele Doblhammer, Rudolf F. Guthoff, Stefanie Frech

**Affiliations:** 1 Institute for Sociology and Demography, University of Rostock, Rostock, Germany; 2 German Center for Neurodegenerative Diseases (DZNE), Bonn, Germany; 3 Department of Ophthalmology, Rostock University Medical Center, Rostock, Germany; Saarland University, GERMANY

## Abstract

**Objective:**

This study aims to estimate the incidence of severe binocular vision impairment and blindness (SVI/B) and to identify eye diseases and regional risk factors of persons with SVI/B at ages 50 years and older.

**Methods:**

We designed an observational cohort study based on longitudinal, multifactorial, and administrative information of a random sample of 250,000 persons at ages 50+. All individuals were included in the process-produced health claims register of the Allgemeine Ortskrankenkasse in 2004, and were followed until 2015. We analyzed ten selected eye diseases and regional characteristics as risk factors for SVI/B using Cox models, adjusting for demographic characteristics and multi-morbidity.

**Results:**

The age-standardized incidence was 79 new diagnoses of SVI/B per 100,000 person-years (95%-CI: 76-82); 77 for males (72-82) and 81 for females (77-85). By adjusting for multiple factors, the model revealed and confirmed that individuals who were very old (Hazard ratio_90+:_ 6.67; 3.59-12.71), male (1.18; 1.01-1.38), had multi-morbidities (three+ diseases: 3.36; 2.51-4.49), or had diabetes (1.26; 1.07-1.49) had an increased risk of SVI/B. Compared to persons without the particular eye disease (all p<0.001), persons diagnosed with secondary glaucoma had a multiple-adjusted 4.66 times (3.17-6.85) higher risk, those with retinal vascular occlusion had a 4.51 times (3.27-6.23) higher risk, and those with angle-closure glaucoma had a 4.22 times (2.60-6.85) higher risk. Population density was not a risk factor, while persons living in wealthier regions had 0.75 times (p=0.003) to 0.70 times (p<0.001) the risk of SVI/B than persons in the least wealthy regions of Germany.

**Conclusion:**

The study revealed and confirmed some profound risk factors of SVI/B at both the individual and the macro level. The sizes of the effects of the characteristics of the living context were smaller than those of the individual characteristics, especially for some severe eye diseases. While urbanity and access to health services had no effect, regional economic wealth was a risk factor for SVI/B. Future health care measures and advice by physicians should take these dimensions of inequalities in SVI/B into account.

## Introduction

The loss of vision leads to severe limitations in quality of life and in independent living. Many studies have investigated the risk factors of selected eye diseases, but only a few studies have analyzed simultaneously the effects of various eye diseases, demographic characteristics, and the characteristics of the regions where the affected individuals live.

Globally in 2015, 36.0 million people were estimated to be blind, and 216.6 million were reported to have moderate to severe vision impairment [1]. As the estimated number of blind people in 1990 was 30.6 million, the number of blind people increased globally by 16.6% between 1990 and 2015 [2]. The leading causes of blindness have been shown to be uncorrected refractive errors, age-related macular degeneration (AMD), glaucoma, and diabetic retinopathy [1]. A common characteristic of these eye diseases is that they worsen with age; therefore, due to population growth and aging, the number of people affected by vision impairment and blindness is increasing [1]. In Europe, the estimated number of blind people aged 50 and older is 1.28 million, while another 9.99 million people have moderate or severe vision impairment. For Germany, the number of blind people has been estimated at 176,190 for 2017, with 43.67% of this population being aged 50 and older [3].

A number of studies for Germany have evaluated the incidence of blindness for individual federal states within the last 30 years: Rohrschneider estimated incidence rates on level of federal states and detected profound differences in SVI/B between the federal states and AMD as the main risk factor of the trends in SVI/B [4]. For the German federal states Baden-Wuerttemberg and Saxony, Claessen and colleagues computed age- and sex-standardized all-cause incidence rates of blindness that decreased over time [5, 6]. These findings based on the analysis of data from social welfare institutions, which are responsible for the distribution of allowances granted for visual impairment; or on data from severely disabled statistics. However, determining the exact numbers is a challenge, as unlike in other countries, there is no national registry of blind people in Germany.

Concerning the risk factors of blindness, AMD is the leading cause in developed countries, with eight million people being blind as a direct result of this disease, according to the WHO [7]. Finger et al. investigated the incidence rates of severe visual impairment and blindness (SVI/B) in 2011, and found that AMD accounted for 50% of all incidence rates with 5.56/100,000 person-years, followed by glaucoma with 1.65/100,000 person-years, and diabetic eye diseases with 1.65/100,000 person-years. Projections for 2030 have estimated that all of these incidence rates will increase at a rate that is twice as high for women as for men [8]. In sum, studies proved various eye diseases like AMD [1, 5, 9], retinopathy including diabetic retinopathy [1, 5], glaucoma including primary open-angle glaucoma, primary angle-closed glaucoma, and secondary glaucoma [1, 5, 9, 10], cataracts including diabetic cataracts [1, 5], myopia [5, 8], retinal vascular occlusion and obliteration [5], disorders of optic nerves and optic atrophy [5, 8], and injuries of the eye [11, 12] to be significant risk factors of SVI/B.

Based on these studies, we selected ten eye diseases in our analysis as risk factors of SVI/B: AMD, retinopathy including diabetic retinopathy, primary open-angle glaucoma, primary angle-closed glaucoma, secondary glaucoma, cataracts including diabetic cataracts, myopia, retinal vascular occlusion and obliteration, disorders of optic nerves, and injuries of the eye.

To learn more about the potential regional differences in the incidence of blindness within Germany, two macro level risk factors were included in our analysis: namely, population density and the available income of private households per capita (economic wealth). We selected these factors because no correlation using these parameters has previously been done for this region. In an earlier study, it was found that among men and women, vision varies with educational status: i.e., for women (over age 30) and for men (between ages 30 and 64), visual difficulties were shown to be significantly more common in the lower educational group than in the upper educational group [13]. No significant differences could be identified between the regions studied. The results suggested that the differences between the educational groups were not just education-specific, but also reflected possible differences in the use of aids or in the supply of appropriate visual aids. There are no studies currently available on this topic Klicken oder tippen Sie hier, um Text einzugeben. The Gutenberg Health Study (GHS), a German population-based cohort study, described a noticeable, but not significant connection of prevalence of visual impairment in the adult population with a lower socio-economic status [14]. Outside of Germany, some studies have reported geographical variations in vision loss linked to socioeconomic status, education, or area deprivation [15–17].

Demographic changes and population aging are expected to lead to an increase in the demand for health care. Therefore, our aim in the present study was to determine the incidence rates of SVI/B, the identification of eye diseases and risk factors of SVI/B, and to associate them with urbanity and regional economic wealth within Germany. Therefore, an observational cohort study based on longitudinal, multifactorial, and administrative information of a random sample of 250,000 persons at ages 50+ was conducted using Cox models, adjusting for demographic characteristics and multi-morbidity. In contrast to previous studies, in which data from social welfare institutions were used to determine visual impairment and blindness, in this study a database from the largest public health insurance fund in Germany was analyzed.

## Materials and methods

### Data

In Germany, the majority of inhabitants (90%) are members of a statutory health insurance and we used data from the largest health insurance fund in Germany, the Allgemeine Ortskrankenkasse (AOK). The data holder provided an age-stratified random sample of 250,000 persons aged 50 and older out of all 25,339,374 insured individuals (30% of the German population) in the first quarter of 2004 [18]. The publicly not accessible complete dataset covered all persons who were living in private households and in institutions like nursing homes. The health claims were recorded quarterly over a period from 2004 to 2015 on an individual level, and included basic information on the person, such as age, sex, the five-digit zip code of the place of residence, and diagnoses from in- and out-patient medical visits using the German ICD-10 classification. The diagnoses were officially registered by the admitting physicians in hospitals (inpatient) and medical practices (outpatient), and were the bases for the financial transfers from the health insurance fund to the physicians and hospitals. In this study, all outpatient and inpatient diagnoses were used to define the patient groups (samples). All diagnoses of the AOK-insured individuals were included until the end of the year 2015, or until they died or changed insurers. Other than the ICD-10 codes, there is no information about the causes of SVI/B in the persons in the sample.

The WIdO (Wissenschaftliches Institut der AOK), the scientific institute of the AOK, granted and approved the access to the data. Due to the current data protection regulations, access to the complete register of all AOK-insured persons was not allowed. The dataset only included anonymized administrative claims data. This study complies with the tenets of the Declaration of Helsinki, and no ethical approval was required.

#### Design of the study

The study used a prospective population-based observational cohort design and longitudinally followed all individuals drawn in the first quarter of 2004. The large sample size and the high number of diagnoses (potential risk factors) permitted us to adjust for and to estimate the effects of multiple risk factors simultaneously. At baseline, we used all insured persons without SVI/B and without any of the eye diseases defined as risk factors.

We followed these individuals until their first diagnosis of SVI/B or exit from the data because of death, a change in insurer, or the end of the observation period. The impact of the incidence eye diseases on the risk of SVI/B was explored.

For each of the eye diseases, we defined the reference groups (also called the control groups) as consisting of those individuals who did not experience this disease. For example, the reference persons for the risk factor myopia were persons without myopia. The model then estimated the risk of being diagnosed with a binocular SVI/B for persons with incident myopia compared to the risk of those without the disease.

Based on the five-digit zip code, we linked the individual data with macro information from the German National Statistical Office on the characteristics of the region.

#### Definition of SVI/B severe vision loss

To investigate the risk of developing SVI/B between different groups of individuals with specific eye diseases (case) and those without (control), we used the ICD-10 code H54.0 for severe binocular visual impairment and blindness (SVI/B, categories 3, 4, and 5).

#### Risk factors and control variables

Based on the literature review, the ten selected eye diseases were primary open-angle glaucoma (H40.1), primary angle-closed glaucoma (H40.2), secondary glaucoma (H40.3-H40.6), myopia (H44.2, H52.1), injuries of the eye (S05, T15, T26), age-related macular degeneration (H35.3), retinopathy (H35.0-H35.2) including diabetic retinopathy (H36.0), cataracts (H25-H26) including diabetic cataracts (H28.0), retinal vascular occlusion (H34), and disorders of optic nerves (H46-H48). Since all eye diseases were assumed to be associated with age, sex, and the general level of multi-morbidity, we adjusted for these factors. The multi-morbidity status was computed as a modified comorbidity index by Charlson et al. (1987), and measured by the quarterly total number of (ever-diagnosed) severe diseases [19]. The selected severe diseases were cerebrovascular diseases (G45-G46, H34.0, I06), ischemic (I20-I25) and other heart diseases (I09.9, I11.0, I13.0-I13.2, I42.0-I42.9, I43, I50), cancer (C00-C97), kidney diseases (I12.0, I13.1-13.2, N03.2-03.7, N05.2-05.7, N11-N19, N25-N29, Z49.0-Z49.2, Z94.0, Z99.2), pneumonia (J12-J18), lung diseases (J44), and dementia (F00.0-00.9, F01.0-01.9, F02.0-02.8, F03, F05.1, G23.1, G30.0-30.9, G31.0, G31.82). We grouped the persons into five categories: none of the severe diseases; and one, two, and three and more of the selected diseases. Due to the importance of diabetes mellitus (E10-E14) as a risk factor for many eye diseases, diabetes mellitus (all types) was analyzed as an independent covariate.

As well as examining the individual-level risk factors, we investigated two macro level risk factors: the degree of urbanization and the economic wealth of the region. The degree of urbanization was measured using the officially calculated population density levels issued by the German National Statistical Office [20]. The population density was defined as the number of persons per square kilometer. The economic wealth indicator was estimated by the same institution based on the income of private households in euros per head. Both indicators were used for the starting year of 2004 for the different counties of Germany, and were assigned to the five-digit zip code regions. Each indicator was categorized into three quantiles at the level of counties. The population density categories were fewer than 130 persons per square km, 130 to 251 persons per square km, and more than 251 persons per square km. The income categories were less than 1,324 euros per head, 1,324 to 1,479 euros per head, and more than 1,479 euros per head. For 2,456 persons, there was no plausible matching of the zip codes; thus, these individuals were classified as having “missing information”, but were included in the regression models.

#### Validation strategy of diagnoses

We used a validation strategy to reduce the probability of false positive diagnoses: i.e., the same diagnoses of a specific eye disease had to be coded in at least two quarters within the observation period (minimum two quarters [M2Q] criterion) [21] for the first diagnosis to be defined as valid. Because of our interest in the incidence of diseases, only valid diagnoses in 2006 to 2015 were considered, while individuals with diagnoses in the years 2004 and 2005 were excluded. In case of the eye diseases, only diagnoses of ophthalmologists were included.

#### Analysis samples

**Fig 1:**
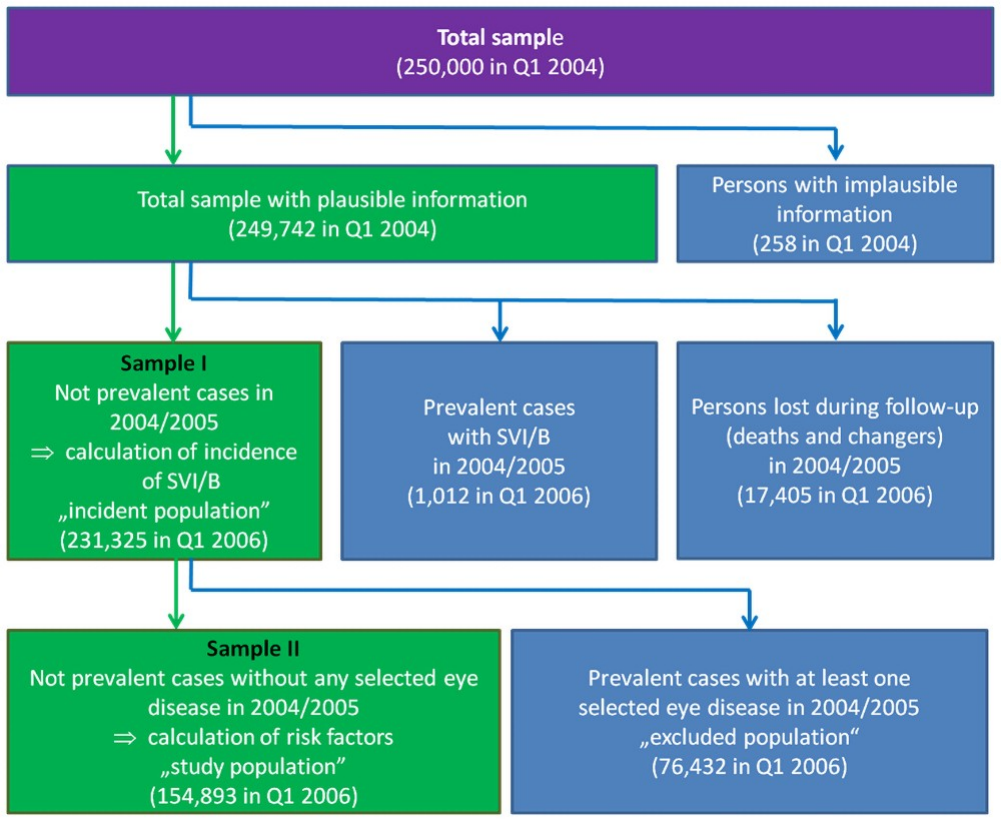
Scheme of analyzed sample compositions (green) and the excluded population (blue), AOK data 2004-2015.

The total sample of 250,000 persons was reduced by 258 persons with implausible information (Fig 1). We also excluded 1,012 persons with validated SVI/B recorded in 2004 and 2005 as prevalent cases for whom the date of incidence was unknown, as well as 17,405 persons who died or changed their insurance in the years 2004 and 2005. This resulting Sample I consisted of 231,325 persons, which we use for the calculation of the incidence of SVI/B. For the multivariable analysis that explores the contribution of different incident eye diseases to this risk, the sample was further reduced by 76,432 persons with at least one of the selected and validated eye diseases diagnosed in 2004 and 2005. The most common prevalent eye diseases diagnosed in 2004 and 2005 in Sample I was cataract in 55,884 persons (24.16% out of 231,325 persons), myopia (18,923 persons; 8.18%), retinopathy (17,259 persons; 7.46%), and age-related macular degeneration (14,090 persons; 6.09%). Rarer diseases with descending prevalence were open-angle glaucoma (9,813 persons, 4.24%), disorders of the optic nerve (8,609 persons; 3.72%), retinal vascular occlusion (1,678 persons; 0.73%), secondary glaucoma (1,373 persons; 0.59%), injuries of the eye (1,286 persons; 0.56%), and angle-closure glaucoma (1,262 persons; 0.55%). These persons were excluded from Sample II.

The resulting Sample II consisted of 154,893 persons aged 50+ without a diagnosis of any of the selected eye diseases at the beginning of the first quarter of 2006. To identify selectivity due to the exclusion from the analysis sample, we also showed the characteristics of the excluded population and made a sensitivity analysis, where persons with prevalent eye diseases were included.

### Statistical methods

#### Incidence rates

We estimated the incidence of SVI/B for all persons from the start of the first quarter of 2006 until the end of the fourth quarter of 2015, or in case of death or of a change in insurance in the following quarters. Person-time under risk was measured in years, and had a total length of 10 years (2006-2015). Thus, the incidence was defined as an annual rate. Sex (x)- and age group (a)-specific incidence rates were defined as the number of persons newly diagnosed with SVI/B between 2006 and the end of 2015 (Inc_2006-15,x,a_) divided by the person-time under risk in years (or person-years, PY_Risk_) from the beginning of 2006 to the end of 2015 (Eq 1).

$${\text{Incidence}}_{2006-15,\text{x},\text{a}}=\frac{{\text{Inc}}_{2006-15,\text{x},\text{a}}}{{\text{PY}}_{\text{Risk},2006-15,\text{x},\text{a}}}∙1000$$ (1)

We calculated the incidence rates by age in five-year age groups (50-54, 55-59, 60-64, 65-69, 70-74, 75-79, 80-84, 85-89, 90+) and sex using Sample I (and Sample II in Appendix). Furthermore, 95% binomial exact confidence intervals were estimated, and the incidence was directly age standardized by using sex- and age-stratified demographic data of the 2004 German population.

#### Incidence of SVI/B by duration of eye diseases

To investigate the effect of the chronology of SVI/B after the incidence of the selected eye diseases, we applied Kaplan-Meier estimators (KME). We computed KME to show the incidence rates of SVI/B at specific time points after the first valid diagnosis of a selected eye disease over the observation period, compared to the incidence rates of SVI/B for persons with other eye diseases or without any eye disease. These individuals were defined as a “control” group. The risk time for a specific eye disease started at the beginning of the first quarter of 2006 for all persons (see technical note in appendix). [22]. In the quarter of the first valid diagnosis of this eye disease, the affected individuals were considered as “cases” with a restart of the time counter at zero. Time was measured in quarters, and ended with the first valid diagnosis of SVI/B, death, a change in insurance, or the end of the fourth quarter of 2015.

#### Risk factors of SVI/B

We applied Cox proportional hazard models to study the simultaneous effects on the risk of developing SVI/B of the incidence of eye disease, demographic factors, multi-morbidity, and macro level factors. In accordance with the estimation of the incidence rates, the risk time started in the first quarter of 2006, and ended with a valid diagnosis of SVI/B, death, a change in insurance, or the end of the last quarter of 2015. The risk time was measured in years, with a maximum of 10 years. The models were simultaneously adjusted for the demographic factors age (50-54, 55-59, 60-64, 65-69, 70-74, 75-79, 80-84, 85-89, 90+) and sex; the health factors multi-morbidity status and diabetes mellitus; the 10 selected eye diseases; and the two macro factors (economic wealth and urbanization). All factors except sex were considered as time-varying. Diseases were defined as chronic, incurable illnesses. Separately for each health problem, persons with no valid diagnosis were indicated as reference groups (“controls”). We did a sensitivity analysis in which all persons with prevalent eye diseases in 2004/05 were included (Sample I) and compared the estimated values.

The results of the models were presented as hazard ratios. A hazard ratio is the ratio of the hazard rate of the cases to the hazard rate of the controls. Since it is a standardized measure, the values of the reference groups were set as one. The results further include p-values and 95%-confidence intervals. We performed all analyses by using Stata version 12.1 (Stata Corp., College Station, Texas, USA).

## Results

### Incidence of SVI/B

In Sample I, we identified 1,991 persons diagnosed with SVI/B out of the 231,325 persons in Sample I. The mean person-years were 7.867 years and the median person-years were 9.875 years. The age-standardized incidence for the total population was 79 new diagnoses of SVI/B per 100,000 person-years (95%-CI: 76-82), with 77 diagnoses for males (95%-CI: 72-82), and 81 diagnoses for females (95%-CI: 77-85). A steep increase in the age-specific incidence was observed at the highest ages for all age groups and both sexes. The incidence was higher for males than for females, but the gender gap was not statistically significant (Table 1). We further estimated the age-specific incidence rates based on the reduced Sample II (see S1 Table in S1 File).

**Table 1:** Incidence of SVI/B (Sample I) by sex and age at incidence, 2006-2015, AOK data.

	**Age**	**Incidence (per 100,000), 95%-CI**		**Cases of SVI/B**		**Person-time**
**Total**	50-54	**30.12**	(*18*.*72-48*.*45*)	17	0.9%	56,442
	55-59	**29.98**	(*23*.*28-38*.*62*)	60	3.0%	200,116
	60-64	**43.52**	(*36*.*25-52*.*25*)	115	5.8%	264,238
	65-69	**53.94**	(*46*.*13-63*.*07*)	157	7.9%	291,074
	70-74	**80.27**	(*71*.*20-90*.*50*)	267	13.4%	332,630
	75-79	**120.93**	(*108*.*88-134*.*31*)	349	17.5%	288,598
	80-84	**195.43**	(*177*.*28-215*.*45*)	404	20.3%	206,719
	85-89	**302.97**	(*273*.*12-336*.*08*)	357	17.9%	117,834
	90+	**484.63**	(*429*.*66-546*.*64*)	265	13.3%	54,681
	**Total**			1,991		1,812,333
	**Age**	**Incidence (per 100,000), 95%-CI**		**Cases of SVI/B**		**Person-time**
**Men**	50-54	**46.06**	(*26*.*75-79*.*33*)	13	1.7%	28,222
	55-59	**32.38**	(*22*.*89-45*.*78*)	32	4.3%	98,843
	60-64	**53.36**	(*42*.*14-67*.*56*)	69	9.2%	129,313
	65-69	**61.49**	(*49*.*71-76*.*05*)	85	11.3%	138,244
	70-74	**92.03**	(*77*.*75-108*.*94*)	135	18.0%	146,690
	75-79	**125.09**	(*106*.*12-147*.*46*)	142	18.9%	113,515
	80-84	**211.56**	(*179*.*37-249*.*53*)	141	18.8%	66,647
	85-89	**270.32**	(*216*.*21-337*.*97*)	77	10.2%	28,485
	90+	**625.46**	(*483*.*54-809*.*03*)	58	7.7%	9,273
	**Total**			752		759,232
	**Age**	**Incidence (per 100,000), 95%-CI**		**Cases of SVI/B**		**Person-time**
**Women**	50-54	**14.17**	(*5*.*32-37*.*77*)	4	0.2%	28,220
	55-59	**27.65**	(*19*.*09-40*.*04*)	28	1.4%	101,273
	60-64	**34.09**	(*25*.*54-45*.*52*)	46	2.3%	134,924
	65-69	**47.11**	(*37*.*39-59*.*35*)	72	3.6%	152,830
	70-74	**70.99**	(*59*.*86-84*.*20*)	132	6.6%	185,940
	75-79	**118.23**	(*103*.*17-135*.*48*)	207	10.4%	175,083
	80-84	**187.76**	(*166*.*39-211*.*88*)	263	13.2%	140,072
	85-89	**313.38**	(*278*.*74-352*.*32*)	280	14.1%	89,350
	90+	**455.87**	(*397*.*81-522*.*40*)	207	10.4%	45,408
	**Total**			1,239		1,053,100

### Characteristics of the study population

At the beginning of the study period, the total study population of Sample II consisted of 154,893 individuals; with the largest age group being ages 65-69 (18.36%) and the smallest age group being ages 90+ (2.40%). The individuals who were newly diagnosed with SVI/B had an older age composition. Out of the 680 persons in this population, the largest share was in the 80-84 age group (17.21%), and the smallest share were in the 50-54 age group (1.76%). The study population and the population with incident SVI/B consisted of slightly more females (54.56% and 56.32%, respectively) than males (45.44% and 43.66%, respectively), while a larger majority of the excluded persons were females (63.95% females versus 36.05% males). When looking at multi-morbidity, most of the study population had no (45.64%) or one severe disease (25.36%), while most of the population with incident SVI/B had two (32.50%) or more than two severe diseases (42.21%). The excluded population had the largest proportion of individuals with two severe diseases (33.71%). There were marked differences between the three populations in the proportions of persons with diabetes mellitus: 15.20% of the total study population had diabetes, while 38.38% of the persons with incident SVI/B and 34.68% of the excluded persons were diagnosed with diabetes. Based on our definition of the study population, no person in the population had a valid diagnosis of any of the selected eye diseases at the beginning of the first quarter of 2006. In the population with incident SVI/B, the most frequent eye diseases were cataracts (51.47%), age-related macular degeneration (23.53%), retinopathy (13.82%), myopia (12.65%), and disorders of the optic nerve (11.76%). The composition of the excluded persons was very similar: the majority (73.12%) was diagnosed with cataracts, while smaller shares were diagnosed with myopia (24.76%), retinopathy (22.58%), age-related macular degeneration (18.43%), and open-angle glaucoma (12.84%).

For the two macro factors we investigated, larger differences were detected for the wealth indicator than for the urbanization indicator. The largest share of the study population were living in highly urbanized regions (37.42%), while the smallest share were living in the least urbanized regions (26.82%). These disparities were similar in the population with incident SVI/B and the excluded population. The contrast between the proportions in the study population and the population with incident SVI/B was greater for household income: 36.52% of the study population and 45.88% of the population with incident SVI/B were living in the regions with the lowest wealth. The proportions for the excluded population correspond to the proportions for the total study population (Table 2).

**Table 2:** Characteristics of the study population of Sample II, the population with incident SVI/B, and the excluded population, 2004-2015, AOK data.

Covariates			Study population Start of first quarter of 2006		Population with incident SVI/B 2006-2015		Excluded population 2004-2005	
**Age**	**50-54**		25,169	16.25%	12	1.76%	3,752	4.91%
	**55-59**		25,161	16.24%	29	4.26%	5,013	6.56%
	**60-64**		23,987	15.49%	61	8.97%	7,299	9.55%
	**65-69**		28,434	18.36%	76	11.18%	13,484	17.64%
	**70-74**		20,157	13.01%	91	13.38%	14,653	19.17%
	**75-79**		14,321	9.25%	110	16.18%	14,267	18.67%
	**80-84**		9,667	6.24%	117	17.21%	10,854	14.20%
	**85-89**		4,279	2.76%	94	13.82%	4,515	5.91%
	**90+**		3,718	2.40%	90	13.24%	2,595	3.40%
**Sex**	**Females**		84,503	54.56%	383	56.32%	48,878	63.95%
	**Males**		70,390	45.44%	297	43.68%	27,554	36.05%
**Multi-morbidity status**	**No severe diseases**		70,687	45.64%	71	10.44%	18,121	23.71%
	**One severe disease**		39,282	25.36%	101	14.85%	19,440	25.43%
	**Two severe diseases**		33,434	21.59%	221	32.50%	25,764	33.71%
	**Three and more severe diseases**		11,490	7.42%	287	42.21%	13,107	17.15%
**Ever Diabetes mellitus**	**No**		131,357	84.80%	419	61.62%	49,928	65.32%
	**Yes**		23,536	15.20%	261	38.38%	26,504	34.68%
**Selected eye diseases (ever diagnosed after 2005)**	**Age related macular degeneration**	**no**	154,893	100.00%	520	76.47%	62,342	81.57%
		**yes**	0	0.00%	160	23.53%	14,090	18.43%
	**Disorders of optic nerve**	**no**	154,893	100.00%	600	88.24%	67,823	88.74%
		**yes**	0	0.00%	80	11.76%	8,609	11.26%
	**Retinopathy**	**no**	154,893	100.00%	586	86.18%	59,173	77.42%
		**yes**	0	0.00%	94	13.82%	17,259	22.58%
	**Myopia**	**no**	154,893	100.00%	594	87.35%	57,509	75.24%
		**yes**	0	0.00%	86	12.65%	18,923	24.76%
	**Retinal vascular occlusions**	**no**	154,893	100.00%	630	92.65%	74,754	97.80%
		**yes**	0	0.00%	50	7.35%	1,678	2.20%
	**Angle-closure glaucoma**	**no**	154,893	100.00%	661	97.21%	75,170	98.35%
		**yes**	0	0.00%	19	2.79%	1,262	1.65%
	**Open-angle glaucoma**	**no**	154,893	100.00%	633	93.09%	66,619	87.16%
		**yes**	0	0.00%	47	6.91%	9,813	12.84%
	**Secondary glaucoma**	**no**	154,893	100.00%	646	95.00%	75,059	98.20%
		**yes**	0	0.00%	34	5.00%	1,373	1.80%
	**Cataract**	**no**	154,893	100.00%	330	48.53%	20,548	26.88%
		**yes**	0	0.00%	350	51.47%	55,884	73.12%
	**Injuries of the eye**	**no**	154,893	100.00%	665	97.79%	75,146	98.32%
		**yes**	0	0.00%	15	2.21%	1,286	1.68%
**Population density**	**Lowest third**		41,540	26.82%	214	31.47%	20,788	27.20%
	**Medium third**		52,761	34.06%	225	33.09%	25,394	33.22%
	**Highest third**		57,968	37.42%	240	35.29%	29,796	38.98%
	**Missing information**		2,624	1.69%	1	0.15%	454	0.59%
**Household income**	**Lowest third**		56,562	36.52%	312	45.88%	30,506	39.91%
	**Medium third**		44,193	28.53%	172	25.29%	21,215	27.76%
	**Highest third**		51,514	33.26%	195	28.68%	24,257	31.74%
	**Missing information**		2,624	1.69%	1	0.15%	454	0.59%
**Total**			**154,893**	**100%**	**680**	**100%**	**76,432**	**100%**

### The probability of not having SVI/B by duration of eye diseases

**Fig 2:**
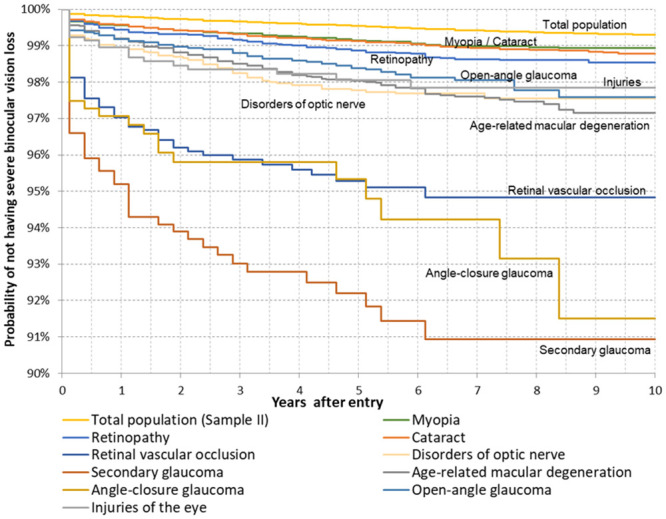
Kaplan-Meier survival functions by eye diseases after the incidence of the eye disease, 2006-2015, AOK data (abbreviated y-axis).

The general probability of not having SVI/B was 99.30% (95%-CI: 99.25-99.34%) for the population with no valid diagnosis of SVI/B after 10 years of observation. The individuals at highest risk of having SVI/B were those with secondary glaucoma, with a proportion of 90.94% (95%-CI: 87.86-93.28%), and with most cases being diagnosed in the first three years after incidence. The individuals with retinal vascular occlusion and angle-closure made up the second-largest risk groups, with very similar trends until year five (both about 95%). Thereafter, the proportion at risk of having SVI/B remained stable for those with retinal vascular occlusion, but was markedly reduced for those with secondary glaucoma (91.51%, 95%-CI: 85.65-95.05%) by the end of the observation period. The individuals with other selected eye diseases had a lower risk of developing SVI/B (Fig 2).

**Fig 3:**
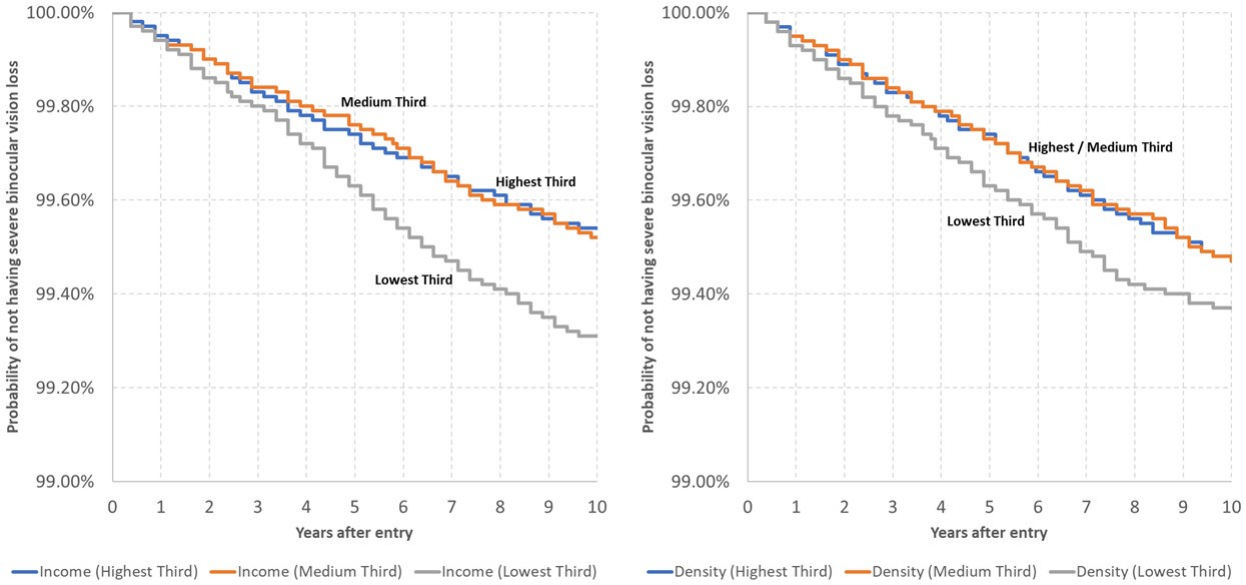
Kaplan-Meier survival functions by type of living region, 2006-2015, AOK data (abbreviated y-axis).

Looking at the macro factors and without adjusting for individual-level characteristics, we see that there were only marginal disparities in the risk of having SVI/B between individuals depending on the type of region where they were living. Nevertheless, the individuals living in the least wealthy regions and those living in the least urbanized regions had a slightly higher risk of developing SVI/B (Fig 3): After 10 years the proportion for individuals in the lowest group of regional wealth was reduced to 99.31% (95%-CI: 99.22-99.38%) and for persons in the lowest group of urbanity was reduced to 99.37% (95%-CI: 99.28-99.45%).

### Risk factors of SVI/B

The multivariable Cox regression model confirmed and uncovered some profound risk factors of SVI/B (Table 3). While simultaneously adjusting for all factors, the risk of developing SVI/B increased steeply after age 80, with individuals aged 90+ having the highest risk. The elderly had a risk of SVI/B that was 6.76 times (p-value<0.001, 95%-CI: 3.59-12.71) higher than that of individuals aged 50-54. Males had a 1.18 times higher risk of SVI/B than females (p=0.042, 95%-CI: 1.01-1.38). People with a high number of severe morbidities were found to have an increased risk of developing SVI/B. For example, compared to persons with none of the selected diseases (p<0.001), individuals with two severe diseases had a risk that was 2.12 times higher (95%-CI: 1.60-2.81), and individuals with three or more severe diseases had a risk that was 3.36 times higher (95%-CI: 2.51-4.49). Having diabetes was associated with a risk of SVI/ that was 1.26 higher (p=0.005, 95%-CI: 1.07-1.49). Consistent with the descriptive analysis of Fig 2, persons with secondary glaucoma, retinal vascular occlusion, and angle-closure glaucoma were found to be at greatest risk of developing SVI/B. Individuals diagnosed with secondary glaucoma had a risk that was 4.66 times higher (95%-CI: 3.17-6.85), those with retinal vascular occlusion had a risk that was 4.51 times higher (95%-CI: 3.27-6.23), and those with angle-closure glaucoma had a risk that was 4.22 times (95%-CI: 2.60-6.85) higher than that of persons without the particular eye disease (p<0.001 for all). Except for retinopathy and open-angle glaucoma, having all other eye diseases was associated with a significantly higher risk of developing SVI/B: Patients with injuries of the eyes had a 2.71 times (95%-CI: 1.62-4.54), patients with AMD had a 2.51 times (95%-CI: 2.04-3.09), patients with disorders of the optic nerve had a 2.17 times (95%-CI: 1.66-2.83), patients with cataract had a 2.08 times (95%-CI: 1.72-2.52) and patients with myopia had a 1.31 times (95%-CI: 1.03-1.66) higher risk of SVI/B.

**Table 3:** Results of the Cox regression models, 2006-2015, Sample II, AOK data.

Covariates			Hazard Ratio	p-value	95% confidence interval
**Age**	**50-54**		**1.00**		
	**55-59**		0.78	0.478	(0.40-1.54)
	**60-64**		1.29	0.434	(0.69-2.41)
	**65-69**		1.25	0.482	(0.67-2.32)
	**70-74**		1.21	0.542	(0.65-2.25)
	**75-79**		1.63	0.121	(0.88-3.02)
	**80-84**		2.40	0.005	(1.30-4.46)
	**85-89**		3.38	<0.001	(1.80-6.34)
	**90+**		6.76	<0.001	(3.59-12.71)
**Sex**	**Females**		**1.00**		
	**Males**		1.18	0.042	(1.01-1.38)
**Multi-morbidity status**	**No severe diseases**		**1.00**		
	**One severe disease**		1.49	0.010	(1.10-2.03)
	**Two severe diseases**		2.12	<0.001	(1.60-2.81)
	**Three and more severe diseases**		3.36	<0.001	(2.51-4.49)
**Ever diabetes mellitus**	**No**		**1.00**		
	**Yes**		1.26	0.005	(1.07-1.49)
**Selected eye diseases (ever diagnosed after 2005)**	**Age related macular degeneration**	**No**	**1.00**		
		**Yes**	2.51	<0.001	(2.04-3.09)
	**Disorders of optic nerve**	**No**	**1.00**		
		**Yes**	2.17	<0.001	(1.66-2.83)
	**Retinopathy**	**No**	**1.00**		
		**Yes**	1.20	0.134	(0.95-1.52)
	**Myopia**	**No**	**1.00**		
		**Yes**	1.31	0.025	(1.03-1.66)
	**Retinal vascular occlusions**	**No**	**1.00**		
		**Yes**	4.51	<0.001	(3.27-6.23)
	**Angle-closure glaucoma**	**No**	**1.00**		
		**Yes**	4.22	<0.001	(2.60-6.85)
	**Open-angle glaucoma**	**No**	**1.00**		
		**Yes**	1.36	0.070	(0.97-1.90)
	**Secondary glaucoma**	**No**	**1.00**		
		**Yes**	4.66	<0.001	(3.17-6.85)
	**Cataract**	**No**	**1.00**		
		**Yes**	2.08	<0.001	(1.72-2.52)
	**Injuries of the eye**	**No**	**1.00**		
		**Yes**	2.71	<0.001	(1.62-4.54)
**Population density**	**Lowest third**		**1.00**		
	**Medium third**		0.95	0.585	(0.78-1.15)
	**Highest third**		0.90	0.314	(0.74-1.10)
	**Missing information**		0.14	0.047	(0.02-0.98)
**Household income**	**Lowest third**		**1.00**		
	**Medium third**		0.70	<0.001	(0.58-0.85)
	**Highest third**		0.75	0.003	(0.62-0.91)
	**Missing information**		-		

The level of population density was not found to be a significant risk factor (p-values>0.05). However, individuals living in wealthier regions had 0.75 times (p=0.003, 95%-CI: 0.62-0.91) to 0.70 times (p<0.001, 95%-CI: 0.58-0.85) the risk of developing SVI/B than those in the least wealthy regions.

### Sensitivity analysis

Sensitivity analysis including all persons diagnosed with any eye disease in 2004/2005 was included show very similar results (S2 Table in S1 File). The age effect was lower, e.g. elders at age 90+ had a 4.89 times (p<0.001, 95%-CI: 2.95-8.13) higher risk of SVI/B compared to persons at age 50-54. Males had a similar effect in both models (hazard ratio 1.12, p=0.022, 95%-CI: 1.02-1.23). The effect of multi-morbidity was slightly reduced. Persons with three and more severe diseases had a 2.85 times (p<0.001, 95%-CI: 2.37-3.42) higher risk compared to persons without severe disease. The effect of diabetes was the same in both regressions (hazard ratio 1.24, p<0.001, 95%-CI: 1.13-1.36). Most differences were in the effects of the eye diseases. For patients with prevalent and incident secondary glaucoma was reduced from a 4.66 times higher (95%-CI: 3.17-6.85) to a 3.31 times higher (95%-CI: 2.81-3.90) risk of SVI/B, the risk for patients with retinal vascular occlusion was reduced from a 4.51 times higher (95%-CI: 3.27-6.23) to a 2.64 times higher (95%-CI: 2.26-3.10), and for patients with angle-closure glaucoma was reduced from a 4.22 times (95%-CI: 2.60-6.85) to a 1.99 times higher (95%-CI: 1.61-2.46). In contrast, the risk of SVI/B increased for patients with age-related macular degeneration from a 2.51 times (95%-CI: 2.04-3.09) higher to a 2.87 times (95%-CI: 2.60-3.18) higher risk and from a 1.36 times (95%-CI: 0.97-1.90) higher risk to a 1.64 times (95%-CI: 1.45-1.84) higher risk of SVI/B in patients with open-angle glaucoma.

## Discussion

The aim of this study was to determine the incidence rates and to analyze the risk factors for developing SVI/B in Germany. To do so, we used data from the largest health insurance fund in Germany.

### Incidence of SVI/B

The age-standardized incidence was 79 per 100,000 person-years, with a steep increase at the highest ages. The incidence of SVI/B has been previously investigated for different federal states of Germany [4]. The incidence has been estimated at 17.5/100,000 inhabitants for Bavaria, 14/100,000 inhabitants for Hesse, 11.6/100,000 inhabitants, 12.3/100,000 inhabitants for Württemberg-Hohenzollern, and 11.1/100,000 person-years for North Rhine-Westphalia Klicken oder tippen Sie hier, um Text einzugeben. A recent study of Claessen and colleagues describes a decrease of the incidence of blindness over the last decade in Saxony. In 2009, the incidence of blindness was 15.7 per 100,000 person years decreasing to 8.9 per 100,000 person years in 2017 [5].

All of these data are based on analyses of data from social welfare institutions, which are responsible for the distribution of allowances granted for visual impairment; or data from the severely disabled statistics. However, determining the exact numbers is a challenge, as unlike in other countries, there is no national registry of blind people in Germany. We found higher incidence rates than these previous estimates. These differences in outcomes likely occurred because under German law, regulated at the level of the federal states (state blindness benefit laws), legal blindness is defined as a reduction of visual acuity to 1/50 (0.02), and severe visual impairment is defined as a visual acuity of 1/20 or less (<0.05). On the one hand, incorrect ICD10 use by physicians could lead to overestimation of SVI/B in health claims data. On the other hand, SVI/B may be underestimated in social assistance data due to (1) lack of awareness that one is eligible for assistance, low incentives for (2) high income groups to apply for assistance because of the need to disclose property for little extra money, for persons with (3) poor language skills, (4) bureaucratic hurdles, (5) need for long-term care and/or living in inpatient facilities, (6) blindness due to accidents (e.g., chemical burns) because these are paid for by workers’ compensation boards. A few studies have analyzed incidence rates in other countries, with conflicting results. Whereas studies from Israel [23], England and Wales [24] described a decrease in the incidence of blindness, a Chinese study reported an increased incidence [25]. The international WHO classification defines blindness using a limit value for visual acuity of 1/20 (0.05, WHO level 3), which is the same level as that for severe visual impairment in Germany. This means that a visual acuity of 0.05 or less includes both severe visual impairment and blindness. Therefore, the differences in the reported incidence rates may be due to the use of these different classification systems and definitions, and a comparison of data remains difficult.

### Risk factors of SVI/B

The study revealed and confirmed significant risk factors for SVI/B at both the individual level and at the level of the macro factors of economic wealth and urbanization. At the individual level, persons aged 90+, persons with multi-morbidities (two or more severe additional diseases), and persons with diabetes had a significant risk of developing SVI/B. The prevalence of eye diseases has been shown to increase with age, especially for the so-called age-related eye diseases, like glaucoma, diabetic retinopathy, and age-related macular degeneration [9]. Moreover, there is evidence that visual impairment is related to a range of other factors that also increase with higher age, such as higher morbidity, an increased risk of falling, increased mortality, and a lower quality of life [26]. To deal with this problem, Machon et al., who also investigated the association between multi-morbidity and vision problems, proposed screening people with a high risk of functional independence loss for cognitive status and vision problems [27]. Findings for Germany in the 1990s indicated that the age-adjusted risk of blindness was around fivefold higher in diabetic individuals than in non-diabetic individuals [28]. In western, industrialized nations, the main causes of diabetic-induced legally defined blindness in working-aged adults have been shown to be proliferative diabetic vitreoretinopathy and diabetic macula edema [29]. A study conducted in Southern Germany that investigated the diabetes-related incidence of blindness found that the incidence in the diabetic population was significantly lower 20 years later than that found in another study that was carried out in 1990 [30]. A similar decrease was also reported by Claessen et al. in 2018 [6]. Although diabetes is still a risk factor for blindness, the incidence has been decreasing, which seems to be mainly attributable to the introduction of disease management programs for diabetes in 2003, the aim of which was to achieve long-term improvements in the clinical outcomes of diabetes patients [31].

The results of the Cox regression models (Table 3) supported previous findings that selected eye diseases such as glaucoma [1, 5, 9, 10], retinal vascular occlusion [5], injuries of the eye [11, 12], and AMD [1, 5, 9] are significant risk factors for SVI/B. Sensitivity analysis further showed that for most of these eye diseases, the risk of SVI/B was much higher in incident (short- and median-time risk) than in prevalent eye diseases (median- and long-time risk).

All of these ophthalmological pathologies are themselves serious eye diseases that could lead to SVI/B. The highest risk was from secondary glaucoma. Secondary glaucoma can result from numerous ocular (e.g., retinal vascular occlusion) and systemic disorders (e.g., hypertension, diabetes). Therefore, an early detection of the primary ocular and systemic diseases that predispose people to developing secondary glaucoma is important to maximize the therapeutic response and to limit the burden of blindness [32, 33]. Primary angle-closure glaucoma (PACG) is estimated to affect approximately 26% of the glaucoma population, and is responsible for nearly half of the cases of glaucoma-related blindness worldwide [34, 35]. It is caused by a sudden increase of intraocular pressure with a disturbance of the aqueous humor flow. Unless this so-called spontaneous glaucoma attack is treated immediately, SVI/B blindness can occur. Age-related macular degeneration (AMD) is one of the leading causes of blindness in the western world [7] and in Germany [6]. Although there is a very high and significant risk of developing SVI/B associated with AMD, AMD is not among the top three risk factors analyzed and found in this study, but among the top five. This might be because of the introduction in 2006 of intravitreal VEGF inhibition, which serves as a long-term or permanent therapy, although there is no difference of ICD-10 codes between wet and dry AMD and to numbers represent both forms. One reason for AMD not being identified as a risk factor of SVI/B in this study might be, that the disease is slowly progressive. The fact that only incident cases were analyzed and prevalent cases were excluded could lead to this finding. A decrease in the incidence of blindness due to the main causes of blindness such as macular degeneration, diabetic retinopathy, and glaucoma during the last two decades was described by Claessen et al. [6].

Two further risk factors investigated in this study were population density and economic wealth. We found that population density had no influence as a risk factor on vision loss, whereas an increased household income/economic wealth significantly decreased the risk of becoming severely visually impaired or blind. Social determinants have been shown to be a major cause of poor health [36]. Having a lower socioeconomic status has been found to be associated with a higher risk of morbidity and mortality [17] and with visual impairment [15, 37, 38]. An association between socioeconomic status and vision loss was also described by Yip et al. in 2014 for the UK [17]. Another study from England examined the geographical variation of blindness and sight impairment. The authors found wide geographical variation in the rates of certification of blindness across England [16]. They concluded that independent of the role of socioeconomic status, the quality of data collection needs to be improved to monitor the number of people with preventable sight loss more closely [16]. In terms of the regional distribution, no differences among women and men without visual impairments could be observed for Germany [13].

### Strengths and limitations of the study

This study has a number of strengths. Since the incidence of SVI/B was relatively low, a long period of observation was necessary for the investigation of the risk factors. The data we used covered a period of 10 years, and a large number of persons diagnosed with various eye diseases, as well as with different combinations of eye diseases. The longitudinal, individual-level design of the routine data allowed us to monitor the health histories, health changes, and etiological pathways of the individuals in our sample. Multivariable Cox regressions were used because these models could detect the risks of eye diseases and macro level factors, while simultaneously adjusting for other individual-level factors. We assume a high validity of the diagnoses, since all diagnoses were recorded evaluations of practicing ophthalmologists and physicians, and were validated based on the M2Q criterion (reduction of false-positive diagnoses). The routine data we used were not affected by self-selected dropouts, reactivity (e.g., social desirability), any response biases or any cognitive biases (e.g., recall bias or overconfidence bias). The direct social and socioeconomic selectivity of access to health services was assumed to be marginal, since the German statutory health insurance system allows visits to ophthalmologists without any extra costs for patients. Furthermore, the study population also included the population in nursing homes, most of whom were not covered by epidemiological surveys.

In addition to these strengths, the study has some limitations. Like all studies with a longitudinal design, there was a growing selection bias due to mortality and changes in insurance with the increasing duration of the observation period. Diagnoses of persons who died or changed their insurance within the observation period were included until the quarter of death or until the change took place, but there was no refreshment of the sample cohort with new individuals after the start of 2004. Moreover, we had no information about the health histories of the individuals before 2004 (problem of left-censoring). As a consequence, eye diseases that were detected earlier or were never detected (false-negative diagnoses) were not considered as risk factors for SVI/B. Furthermore, the health claims data did not cover the causes of SVI/B. Thus, we were only able to estimate the risk of developing SVI/B of patients with selected eye diseases, but not caused by these diseases. Thus, the causal pathways had to be stated with caution. Because there was no information on the causes of SVI/B in the health claims, we selected eye diseases as risk factors by combining the results of previous studies. This selection is assumed to be incomplete, which is another limitation of the study. During data preparation, we defined some exclusion criteria. One main criterion was to exclude all prevalent persons with any of the selected eye diseases diagnosed in 2004 and 2005. The strategy excluded persons with SVI/B before 2006 and persons with medium-term and long-term duration of eye diseases. This may underestimate the impact of diseases with high risk of SVI/B in early and midlife and the impact of slowly progressing diseases. While we were unable to quantify the first bias, we performed sensitivity analysis to investigate the second bias. Sensitivity analysis showed similar but less extreme effects for most eye diseases, which indicated only a small bias.

Merging macro-level data with individual-level information is another source of problems. On the one hand, the strategy misleads us to interpret macro-level factors (e.g., regional household income per capita) as individual-level factors (e.g., individual household income). On the other hand, the choice of spatial aggregation level and units is problematic [39]. Given the quality of the harmonized data, we chose county-level data; however, data on smaller units such as neighborhoods and municipalities could reveal further disparities and associations if there were a reliable data source.

As mentioned above, another limitation was the definition of vision loss. The health information of the health claims was measured using ICD-10 codes. In comparison to other studies, an overestimation may have been possible, as the ICD-10 code that was the basis of the analysis included both severe visual impairment (visual acuity <0.05) and blindness (visual acuity of 0.02 or less). Although ICD-10 is an international coding system, coding practices have varied between countries and over time. Additionally, the definitions and diagnostic measurements used in ophthalmological surveys or clinical trials may have differed. We had no information about how the eye diseases were diagnosed by the ophthalmologists, or any opportunity to directly validate them retrospectively. These differences reduced the comparability of the incidence rate and of the regression results. The selection of eye diseases and of the other risk factors and the interaction of the risk factors can also be discussed. The last limitation is linked to the nature of the register. The results, which were based on a sample from Germany’s largest health insurance provider AOK covering about 30% of the German population and about 37% of all statutory insured persons, were representative of the AOK population [18]. Compared to the total German population, the AOK members were, on average, of a lower socioeconomic status and older, [40]. However in case of mortality, there was no big difference between AOK data and vital statistics [41].

To our knowledge, this study is the first that has simultaneously investigated selected risk factors for SVI/B at the individual and the macro level by using a large-scale health dataset and established statistical methods. The study uncovered various significant risk factors at both levels. However, in comparing the estimated hazards of the factors, the effects of the characteristics of the place of residence were found to be of less importance than the effects of the personal characteristics. The study showed that factors related to infrastructure or access to the health care system seemed to have no effect for Germany, whereas regional economic wealth was found to be a risk factor for SVI/B, even in a welfare state with an egalitarian health care system like Germany. Further investigations are needed to determine whether similar effects also apply to people living in other welfare states.

## Supporting information

S1 File (DOCX)
