# Supplementary material for: Incidence, individual, and macro level risk factors of severe binocular visual impairment and blindness in persons aged 50 and older
Source: PLoS One. 2021 May 3;16(5):e0251018. doi: 10.1371/journal.pone.0251018 (PMC8092648; doi:10.1371/journal.pone.0251018)
Supplement: S1 File — (DOCX) [file pone.0251018.s001.docx]

**S1 File**

S1 Table: Incidence of SVI/B by sex and age at incidence of SVI/B, (Sample II), 2006-2015, AOK data

S2 Table: Results of the Cox regression models, Sample II, 2006-2015, AOK data

***Additional information about the data sample(s):***

***Exits of the study population over observation time:*** Sample II experienced various transitions over the observation period that led to an exit (E) from the study. The cohort started with 154,893 persons without any selected eye disease or SVI/B. At each quarter, persons of this group were E1) diagnosed with SVI/B, E2) died, E3) changed insurance, or E4), or none of these (right censored) (Figure 4 and Figure 5). In 2006, for example, 5,965 persons died, 4,466 persons changed insurance, and 101 persons were diagnosed with SVI/B. Until the end of 2015, 39,205 persons died in previous years, 3,975 died in 2015, 13,300 previously changed insurance, 1,444 changed insurance in 2015, 655 were previously diagnosed with SVI/B, and 25 were newly diagnosed with SVI/B in 2015. Meanwhile, 96,289 persons were right-censored in 2015.

S1 Fig.: Change in the study population over the observation period (numbers of [former] incidence population not shown)


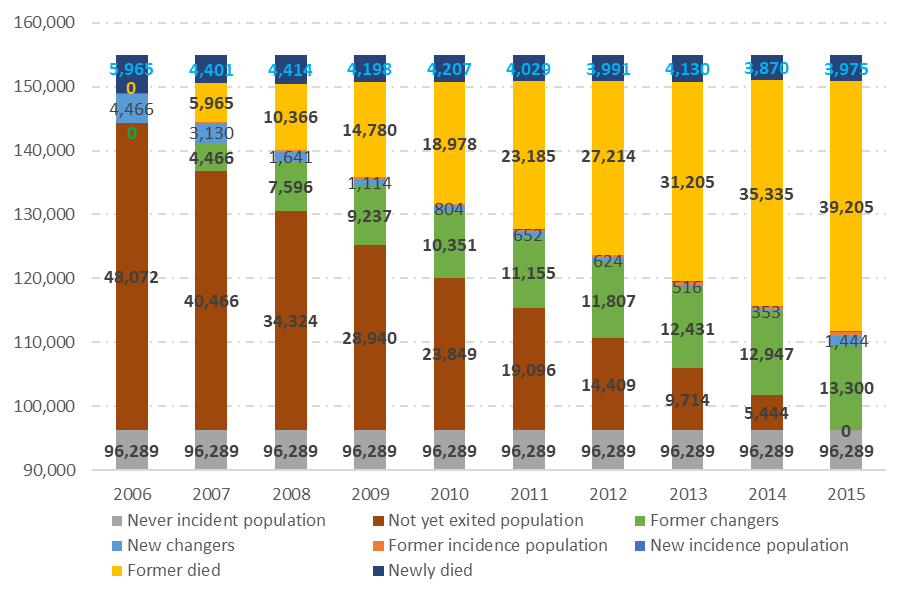


S2 Fig.: Former and new incidence cases over the observation period


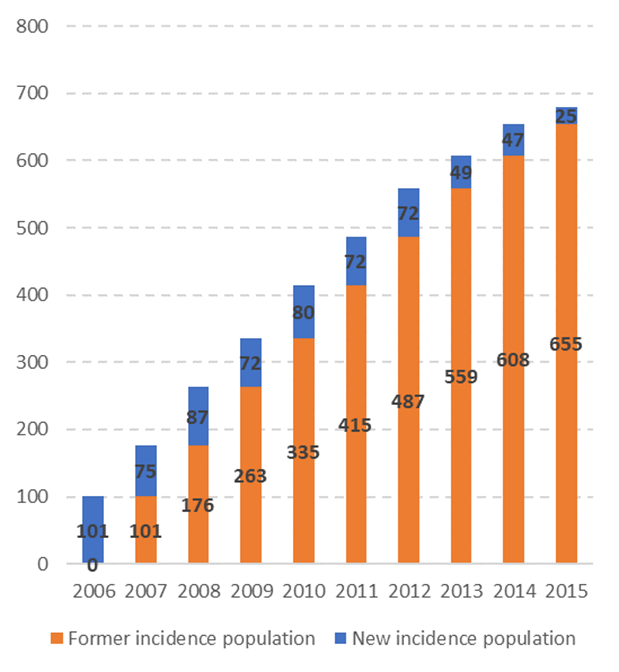


***Observation duration by subgroups:*** Over the whole period, 680 out of the 154,893 persons (0.4%) were newly diagnosed with SVI/B, 43,180 persons (27.9%) died before receiving a valid diagnosis, 14,744 persons (9.5%) changed insurance before a diagnosis, and 96,289 persons (62.2%) were right-censored. Within the eye disease subgroups, the highest proportions of new diagnoses of SVI/B were for secondary glaucoma (5.9%), retinal vascular occlusion (4.1%), and angle-closure glaucoma (4.0%). The highest proportions of deaths were reported for persons with injuries of the eyes (47.3%) and with secondary glaucoma (34.5%). Consequently, the proportion of right-censored persons was lower for these groups of patients. There were no marked differences in the proportions of individuals who changed insurance between the subgroups.

On average, myopia was diagnosed 3.36 years after the entry into the study (earliest diagnosis), while angle-closure glaucoma was diagnosed 4.56 years thereafter (latest diagnosis). The mean time at risk for the total sample was 7.93 years. Hence, persons with angle-closure glaucoma had the shortest total mean time at risk (4.33 years), and persons with myopia had the longest time (5.76 years). The average risk time within the cohort until a new diagnosis of SVI/B was 4.42 years (later than 2005). The shortest mean time to developing SVI/B was found for persons with retinal vascular occlusions (1.25 years) and secondary glaucoma (1.59 years). Persons with open-angle glaucoma (2.62 years) and patients with retinopathy (2.51 years) had the longest mean time of risk until developing SVI/B (Table 3).

S3 Table: Statistics of observation duration by causes of exits and by eye disease, 2006-2015, AOK data

**Technical note:**

In contrast to the definition of risk time for the incidence rates, risk time was defined differently for A) each specific eye disease and B) for persons with (cases) and without this specific eye disease (control). To avoid the immortal (or risk-free) time bias, a general problem of an observational study; we emulated a randomized target trial by a differentiated definition of the risk time.
